# A new organic–inorganic compound, ethyl­enedi­ammonium hexa­chlorido­stannate(IV) *p*-anisaldehyde disolvate

**DOI:** 10.1107/S205698902100579X

**Published:** 2021-06-08

**Authors:** Adrienne Ndiolene, Tidiane Diop, Mouhamadou Sembène Boye, Aminata Diasse-Sarr, Ulli Englert

**Affiliations:** a Laboratoire de Chimie Minérale et Analytique, Département de Chimie, Faculté des Sciences et Techniques, Université Cheikh Anta Diop, Dakar, Senegal; b Département de Physique Chimie, Faculté des Sciences et Technologies de l’Education et de la Formation, Université Cheikh Anta Diop, Boulevard Habib, Bourguiba, BP 5036 Fann-Dakar, Senegal; c Institute of Inorganic Chemistry, RWTH Aachen University, Landoltweg 1, 52056 Aachen, Germany

**Keywords:** crystal structure, hexa­chlorido­stannate(IV) complex, ethyl­enedi­ammonium, *p*-anisaldehyde, organic–inorganic hybrid complex

## Abstract

The asymmetric unit of the title organic–inorganic hybrid complex [systematic name: ethane-1,2-diaminium hexa­chlorido­stannate(IV)–4-meth­oxy­benz­alde­hyde (1/2)], (C_2_H_10_N_2_)[SnCl_6_]·2C_8_H_8_O_2_, contains one half of an ethyl­enedi­ammonium cation, one half of an [SnCl_6_]^2−^ anion and one *p*-anisaldehyde mol­ecule. Both the organic cation and the quasi-regular octa­hedral inorganic anion are located about inversion centres. The organic cations and [SnCl_6_]^2−^ anions lie in layers parallel to the *ac* plane with *p*-anisaldehyde mol­ecules occupying the space between the layers. A network of classical N—H⋯Cl and N—H⋯O hydrogen bonds exists between the ethyl­enedi­ammonium cations and the [SnCl_6_]^2−^ anions and *p*-anisaldehyde mol­ecules. These inter­actions, together with non-classical C—H⋯O inter­actions between the ethyl­enedi­ammonium cations and the *p*-anisaldehyde mol­ecules, serve to hold the structure together. The crystal studied was refined as a two-component twin.

## Abstract 2

This new organic–inorganic hybrid compound contains stacked sheets of ethyl­endi­ammonium cations and [SnCl_6_]^2−^ anions with *p*-anisaldehyde mol­ecules occupying occupying the space in between.

## Chemical context   

The combination of organic and inorganic components to form organic–inorganic hybrid materials has attracted considerable attention owing to the generation of new properties that are absent in type either of building block (Boopathi *et al.*, 2017 ▸; Newman *et al.*, 1989 ▸; Chun & Jung, 2009 ▸; Bouchene *et al.*, 2018 ▸). Hybrid functional materials, containing both inorganic and organic components, are considered to be potential platforms for applications in extremely diverse fields, such as optics, micro-electronics, magnetism, vibrational spectroscopy, transportation, health, energy, energy storage, diagnosis, housing and the environment (Masteri-Farahani *et al.*, 2012 ▸; Kim *et al.*, 2020 ▸; Manser *et al.*, 2016 ▸; Rademeyer *et al.*, 2007 ▸). Moreover, halogenostannate hybrid compounds containing protonated amine cations have recently received considerable attention because of their inter­esting physical and chemical properties, such as magnetism, electroluminescence, photoluminescence and conductivity, which may lead to technological innovations (Aruta *et al.*, 2005 ▸; Chouaib & Kamoun, 2015 ▸; Papavassiliou *et al.*, 1999 ▸; Yin & Yo, 1998 ▸). The structures of these hybrid materials have been shown to contain contain isolated or connected chains or clusters of Sn*X*_6_ octa­hedra separated by amine cations (Zhou & Liu, 2012 ▸; Shahzadi *et al.*, 2008 ▸; Liu, 2012 ▸; Diop *et al.*, 2020 ▸). In this category of materials, the organic moieties, which balance the negative charge on the inorganic units, may also act as structure-directing agents and greatly affect the structure and dimensionality of the supra­molecular framework formed (Díaz *et al.*, 2006 ▸; Hannon *et al.*, 2002 ▸). In the present study, we report the synthesis and structural analysis of a new organic–inorganic hybrid complex, (C_2_H_10_N_2_)[SnCl_6_]·2C_8_H_8_O_2_.
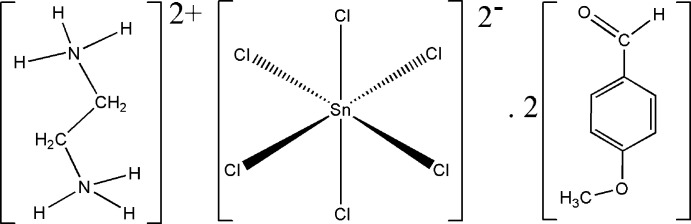


## Structural commentary   

**Figure 1:**
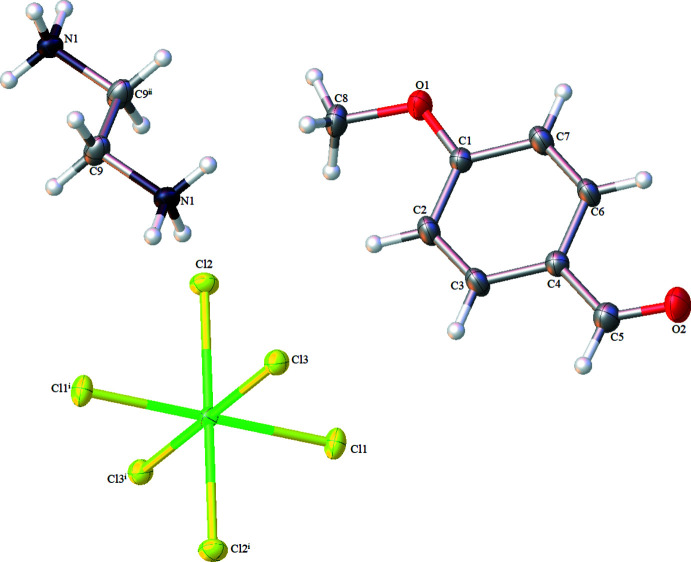
The atom-numbering for the asymmetric unit of the title compound. Displacement ellipsoids are drawn at the 50% probability level. [Symmetry codes: (i) −*x*, −*y*, −*z*; (ii) −*x* + 1, −*y*, −*z* + 1.]

The asymmetric unit comprises of one half of an ethyl­enedi­ammonium cation, one half of a hexa­chloro­stannate(IV) dianion, [SnCl_6_]^2−^, both of which lie on centres of inversion, and one mol­ecule of *p*-anisaldehyde (Fig. 1 ▸). The environment around the tin atom in the [SnCl_6_]^2−^ dianion is an almost undistorted octa­hedron in which the Sn—Cl bond lengths lie in the range 2.4100 (12) to 2.4322 (11) Å and the *cis* Cl—Sn—Cl bond angles lie in the range 89.36 (4) to 90.20 (4) °. The Sn—Cl2 bond involved in hydrogen bonding is slightly longer, at 2.4322 (11) Å, than the other Sn—Cl bonds [Sn—Cl1 = 2.4100 (12)Å and Sn—Cl3 = 2.4220 (11) Å]. These results are comparable to those reported by other research groups (van Megen *et al.*, 2013 ▸; Ali *et al.*, 2008 ▸; Xue & Kong 2014 ▸).

## Supra­molecular features   

**Figure 2:**
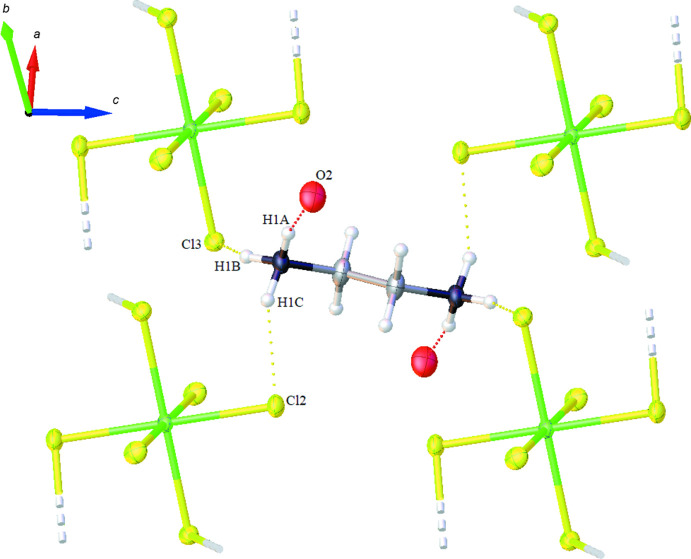
The arrangement of the (C_2_H_10_N_2_)^2−^ and [SnCl_6_]^2−^ units of the title compound in the *ac* plane showing the N1—H1*C*⋯Cl2 and N1—H1*A*⋯O2 hydrogen bonds as dashed lines.

**Figure 3:**
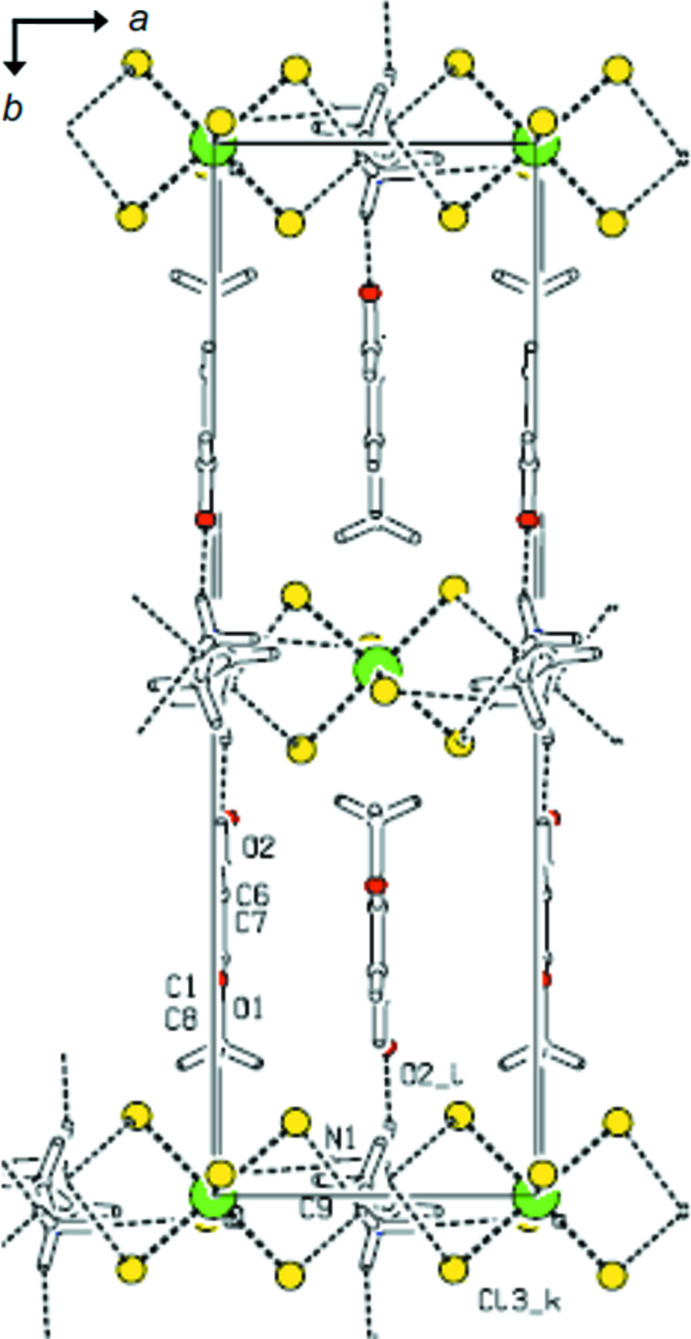
View of the title compound along the *c* axis showing the organic cation–inorganic anion layers separated by *p*-anisaldehyde mol­ecules. Hydrogen bonds are indicated by dashed lines.

The packed crystal structure contains sheets lying parallel to the *ac* plane in which each [SnCl_6_]^2−^ dianion is surrounded by four ethyl­enedi­ammonium cations (Fig. 2 ▸). The *p*-anisaldehyde mol­ecules are located in the otherwise empty space between the sheets (Fig. 3 ▸). The crystal packing of the complex is supported by N—H⋯Cl and N—H⋯O hydrogen-bonding inter­actions (Table 1 ▸). The NH_3_^+^ groups of the ethyl­enedi­ammonium cation act as the hydrogen-bonding donors. The *D⋯A* distances involving the NH_3_^+^ group and either the *p*-anisaldehyde mol­ecule or the [SnCl_6_]^2−^ units range from 2.763 (6) Å for N1⋯O2^iii^ to 3.404 (4) Å for N1⋯Cl3^v^. Non-classical inter­actions between the *p*-anisaldehyde mol­ecules and the ethyl­enedi­ammonium cations, C9—H9⋯O2^vi^ at 2.62 Å, further serve to hold the structure together.

## Database survey   

Organic–inorganic hybrid compounds with structures most similar to that of the title compound include: (C_6_H_22_N_4_)[SnCl_6_]Cl_2_·2H_2_O and (C_8_H_24_N_4_)[SnCl_6_]Cl_2_·2H_2_O (Bouchene *et al.* 2018 ▸), (C_5_H_5_BrN_2_)[SnCl_6_] (Ali *et al.*, 2008 ▸), (C_5_H_7_N_2_)_2_[SnCl_6_], and (C_7_H_10_N)_2_[SnCl_6_] (Rademeyer *et al.*, 2007 ▸) and (C_8_H_12_N)_3_SnBr_6_·Br (Chouaib & Kamoun, 2015 ▸). These structures contain isolated or connected chains or clusters of Sn*X*_6_ octa­hedra separated by the organic cations. A variety of inter­molecular hydrogen bonds, N—H⋯O, N—H⋯Cl and O—H⋯O, together with C—H⋯π inter­actions, serve to consolidate the mol­ecular structures.

## Synthesis and crystallization   

Chemicals [*p*-anisaldehyde, ethyl­enedi­amine and tin(II)] were purchased from Sigma-Aldrich and were used without any further purification. The solvent use for the synthesis was ethanol (96%).

**Synthesis of*****N***,***N*****′-bis­(4-meth­oxy­benzyl­idene)ethyl­enedi­amine**

**Figure 4:**
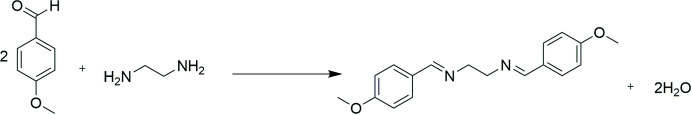
Synthesis of the inter­mediate *N*,*N*′-bis­(4-meth­oxy­benzyl­idene)ethyl­enedi­amine.

The Schiff base *N*,*N*′-bis­(4-meth­oxy­benzyl­idene)ethyl­enedi­amine was prepared by condensing *p*-anisaldehyde (10 g; 0.0734 mol) with ethyl­enedi­amine (2.205 g; 0.0367 mol) in ethanol (30 ml) (Fig. 4 ▸). The resulting mixture was heated under reflux for 6 h, filtered and left to evaporate at ambient temperature. (The reaction between *p*-anisaldehyde and ethyl­enedi­amine gave the same product whatever the proportions of reactants used). After a few days of slow evaporation, 4.511 g of crystals were obtained, corresponding to a yield of 82%. The compound was characterized by FT–IR (cm^−1^: 1639.05 (C=N); 1603, 1505, 1461 and 1448 (C=C, aromatic); 1019 (C—O, ether).

**Synthesis of the title compound**

**Figure 5:**
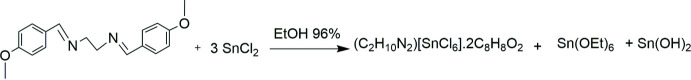
Synthesis of the title compound.

0.3 g (0.00168 mol) of *N*,*N*′-bis (4-meth­oxy­benzyl­idene)ethyl­enedi­amine were dissolved in 30 ml of ethanol in a round-bottomed flask, followed by the addition of SnCl_2_ (0.638 g; 0.00168 mol) to form a yellow solution (Fig. 5 ▸). The mixture was refluxed for 7 h at 353 K, filtered to remove Sn(OEt)_6_ and Sn(OH)_2_ and the resulting solution was allowed to evaporate slowly. After a few days of evaporation, light-yellow block-shaped crystals suitable for single-crystal X-ray analysis were obtained in a yield of 31%. The presence of water mol­ecules in the solvent (EtOH, 96%) causes hydrolysis of the Schiff base and oxidation of tin(II) to tin(IV). The hydrolysis reaction leads to the formation of two mol­ecules of *p*-anisaldehyde and one ethyl­enedi­ammonium cation.

The crystalline product was characterized by FT–IR (cm^−1^: 1659 (C=O); 3290 (N—H); 2801 (C—H, aldehyde); 1596, 1570 and 1556 (C=C, phen­yl); 1259 (C—O, ether).

## Refinement   

Crystal data, data collection and structure refinement details are summarized in Table 2 ▸. (C_2_H_10_N_2_)[SnCl_6_]·2C_8_H_8_O_2_ crystallizes in the space group *P*2_1_/*n* with the monoclinic angle, β, close to 90°. The crystals formed as non-merohedral twins with about one quarter of reflections overlapping. The twin law corresponds to rotation about c*. For the crystal investigated, the relative domain sizes amounted to 0.790 (4): 0.210 (4). The structure was solved by intrinsic phasing (Sheldrick, 2015*a* ▸). The twin law was identified from reflections with *I*_obs_ >> *I*_calc_, and *PLATON* (Spek, 2020 ▸) was used to generate a suitable two-domain reflection file for twin refinement (Sheldrick, 2015*b* ▸). All non-hydrogen atoms were assigned anisotropic displacement parameters. H atoms attached to C were calculated in standard geometry and treated as riding [C—H = 0.95–0.99 Å; *U*_iso_(H) = 1.2*U*_iso_(C) or 1.5*U*_iso_(C-meth­yl)]. H atoms attached to N were located as local maxima in a difference-Fourier map and refined with a distance restraint N—H = 0.9 Å and an isotropic displacement parameter *U*_iso_(H) = 1.2*U*_iso_(N).

## Supplementary Material

Crystal structure: contains datablock(s) I. DOI: 10.1107/S205698902100579X/cq2042sup1.cif

Structure factors: contains datablock(s) I. DOI: 10.1107/S205698902100579X/cq2042Isup2.hkl

CCDC reference: 2063269

Additional supporting information:  crystallographic information; 3D view; checkCIF report

## Figures and Tables

**Table 1:** Hydrogen-bond geometry (Å, °)

*D*—H⋯*A*	*D*—H	H⋯*A*	*D*⋯*A*	*D*—H⋯*A*
C6—H6⋯Cl1^i^	0.95	3.05	3.596 (5)	118
N1—H1*A*⋯O2^i^	0.90 (2)	1.89 (3)	2.763 (6)	162 (6)
N1—H1*B*⋯Cl1^ii^	0.92 (2)	2.71 (5)	3.312 (4)	124 (5)
N1—H1*B*⋯Cl3^iii^	0.92 (2)	2.62 (4)	3.404 (4)	144 (5)
N1—H1*C*⋯Cl2	0.92 (2)	2.44 (3)	3.315 (5)	158 (6)
N1—H1*C*⋯Cl3	0.92 (2)	2.75 (6)	3.292 (4)	119 (5)
C9—H9*B*⋯O2^iv^	0.99	2.62	3.319 (7)	128

Symmetry codes: (i) x+{\script{1\over 2}}, -y+{\script{1\over 2}}, z+{\script{1\over 2}}; (ii) x+1, y, z; (iii) -x+1, -y, -z; (iv) -x+{\script{1\over 2}}, y-{\script{1\over 2}}, -z+{\script{1\over 2}}.

**Table 2:** Experimental details

Crystal data	
Chemical formula	(C_2_H_10_N_2_)[SnCl_6_]·2C_8_H_8_O_2_
*M*_r_	665.80
Crystal system, space group	Monoclinic, *P*2_1_/*n*
Temperature (K)	100
*a*, *b*, *c* (Å)	6.9762 (12), 22.806 (4), 8.0394 (13)
β (°)	90.948 (4)
*V* (Å^3^)	1278.9 (4)
*Z*	2
Radiation type	Mo *K*α
μ (mm^−1^)	1.65
Crystal size (mm)	0.17 × 0.17 × 0.13

Data collection	
Diffractometer	Bruker D8 gonimeter with APEX CCD detector
Absorption correction	Multi-scan (*SADABS*; Krause *et al.*, 2015 ▸)
No. of measured, independent and observed [*I* > 2σ(*I*)] reflections	3929, 3929, 3182
*R*_int_	0.112
(sin θ/λ)_max_ (Å^−1^)	0.723

Refinement	
*R*[*F*^2^ > 2σ(*F* ^2^)], *wR*(*F* ^2^), *S*	0.062, 0.164, 1.07
No. of reflections	3929
No. of parameters	154
No. of restraints	3
H-atom treatment	H atoms treated by a mixture of independent and constrained refinement
Δρ_max_, Δρ_min_ (e Å^−3^)	2.91, −2.69

Computer programs: *SMART* (Bruker, 2002 ▸), *SAINT* (Bruker, 2009 ▸), *SHELXT* (Sheldrick 2015*a* ▸), *SHELXL2018/3 (Sheldrick, 2015*b* ▸)* and *PLATON* (Spek, 2020 ▸).

## supplementary crystallographic information

### Crystal data

**Table:**

(C_2_H_10_N_2_)[SnCl_6_]·2C_8_H_8_O_2_	*F*(000) = 664
*M**_r_* = 665.80	*D*_x_ = 1.729 Mg m^−^^3^
Monoclinic, *P*2_1_/*n*	Mo *K*α radiation, λ = 0.71073 Å
*a* = 6.9762 (12) Å	Cell parameters from 4273 reflections
*b* = 22.806 (4) Å	θ = 2.7–27.3°
*c* = 8.0394 (13) Å	µ = 1.65 mm^−^^1^
β = 90.948 (4)°	*T* = 100 K
*V* = 1278.9 (4) Å^3^	Block, light yellow
*Z* = 2	0.17 × 0.17 × 0.13 mm

### Data collection

**Table:**

Bruker D8 gonimeter with APEX CCD detector diffractometer	3929 independent reflections
Radiation source: Incoatec microsource	3182 reflections with *I* > 2σ(*I*)
Multilayer optics monochromator	*R*_int_ = 0.112
ω scans	θ_max_ = 30.9°, θ_min_ = 1.8°
Absorption correction: multi-scan (SADABS; Krause *et al.*, 2015)	*h* = −9→9
	*k* = −32→32
3929 measured reflections	*l* = −11→11

### Refinement

**Table:**

Refinement on *F*^2^	Hydrogen site location: mixed
Least-squares matrix: full	H atoms treated by a mixture of independent and constrained refinement
*R*[*F*^2^ > 2σ(*F*^2^)] = 0.062	*w* = 1/[σ^2^(*F*_o_^2^) + (0.090*P*)^2^ + 3.*P*] where *P* = (*F*_o_^2^ + 2*F*_c_^2^)/3
*wR*(*F*^2^) = 0.164	(Δ/σ)_max_ < 0.001
*S* = 1.07	Δρ_max_ = 2.91 e Å^−^^3^
3929 reflections	Δρ_min_ = −2.69 e Å^−^^3^
154 parameters	Extinction correction: SHELXL-2018/3 (Sheldrick 2015b), Fc^*^=kFc[1+0.001xFc^2^λ^3^/sin(2θ)]^-1/4^
3 restraints	Extinction coefficient: 0.0073 (13)
Primary atom site location: dual	

### Special details

**Table:**

Geometry. All esds (except the esd in the dihedral angle between two l.s. planes) are estimated using the full covariance matrix. The cell esds are taken into account individually in the estimation of esds in distances, angles and torsion angles; correlations between esds in cell parameters are only used when they are defined by crystal symmetry. An approximate (isotropic) treatment of cell esds is used for estimating esds involving l.s. planes.
Refinement. Refined as a two-component twin.

### Fractional atomic coordinates and isotropic or equivalent isotropic displacement parameters (Å^2^)

**Table:**

	*x*	*y*	*z*	*U*_iso_*/*U*_eq_	
O1	0.0054 (6)	0.20471 (16)	0.5640 (5)	0.0297 (8)	
O2	0.0223 (7)	0.35799 (17)	−0.0922 (5)	0.0346 (9)	
C1	0.0073 (7)	0.2253 (2)	0.4064 (6)	0.0216 (9)	
C2	0.0110 (8)	0.1905 (2)	0.2647 (6)	0.0271 (11)	
H2	0.011776	0.148942	0.273751	0.032*	
C3	0.0134 (8)	0.2171 (2)	0.1100 (6)	0.0261 (10)	
H3	0.014641	0.193476	0.012738	0.031*	
C4	0.0141 (8)	0.2776 (2)	0.0945 (6)	0.0224 (9)	
C5	0.0197 (8)	0.3047 (2)	−0.0684 (7)	0.0289 (11)	
H5	0.021414	0.279678	−0.162937	0.035*	
C6	0.0097 (8)	0.3121 (2)	0.2377 (6)	0.0262 (10)	
H6	0.008962	0.353603	0.227991	0.031*	
C7	0.0063 (9)	0.2865 (2)	0.3939 (7)	0.0281 (11)	
H7	0.003288	0.310129	0.491121	0.034*	
C8	0.0077 (9)	0.1422 (2)	0.5874 (7)	0.0299 (11)	
H8A	−0.104360	0.124858	0.531118	0.045*	
H8B	0.003967	0.133301	0.706506	0.045*	
H8C	0.124940	0.125913	0.540404	0.045*	
Sn1	0.000000	0.000000	0.000000	0.01522 (16)	
Cl1	−0.23806 (17)	0.07591 (5)	−0.03230 (15)	0.0231 (3)	
Cl2	0.02718 (18)	0.02031 (5)	0.29645 (13)	0.0225 (3)	
Cl3	0.25385 (17)	0.06982 (5)	−0.05138 (14)	0.0207 (3)	
N1	0.5008 (6)	0.02942 (18)	0.2818 (5)	0.0209 (8)	
H1A	0.533 (8)	0.0662 (13)	0.313 (8)	0.025*	
H1B	0.544 (9)	0.015 (3)	0.183 (5)	0.025*	
H1C	0.372 (4)	0.029 (3)	0.255 (8)	0.025*	
C9	0.5429 (9)	−0.0111 (2)	0.4218 (6)	0.0259 (11)	
H9A	0.683408	−0.014942	0.437370	0.031*	
H9B	0.490162	−0.050389	0.395586	0.031*	

### Atomic displacement parameters (Å^2^)

**Table:**

	*U*^11^	*U*^22^	*U*^33^	*U*^12^	*U*^13^	*U*^23^
O1	0.049 (2)	0.0189 (17)	0.0210 (17)	0.0022 (16)	−0.0011 (16)	0.0025 (13)
O2	0.052 (3)	0.0207 (18)	0.031 (2)	−0.0010 (17)	0.0017 (18)	0.0066 (15)
C1	0.030 (2)	0.016 (2)	0.019 (2)	0.0003 (18)	−0.0028 (18)	0.0013 (16)
C2	0.043 (3)	0.014 (2)	0.025 (2)	0.001 (2)	−0.001 (2)	−0.0011 (17)
C3	0.041 (3)	0.015 (2)	0.022 (2)	−0.001 (2)	0.001 (2)	−0.0024 (16)
C4	0.030 (2)	0.017 (2)	0.020 (2)	−0.0004 (18)	−0.0019 (18)	0.0007 (16)
C5	0.040 (3)	0.022 (2)	0.024 (2)	0.000 (2)	−0.001 (2)	0.0026 (19)
C6	0.042 (3)	0.014 (2)	0.023 (2)	0.000 (2)	−0.001 (2)	−0.0024 (17)
C7	0.044 (3)	0.016 (2)	0.024 (2)	0.000 (2)	0.000 (2)	−0.0026 (17)
C8	0.043 (3)	0.018 (2)	0.029 (3)	0.003 (2)	−0.002 (2)	0.0067 (18)
Sn1	0.0197 (3)	0.0131 (2)	0.0128 (2)	−0.00059 (15)	−0.00159 (15)	0.00193 (13)
Cl1	0.0246 (6)	0.0173 (5)	0.0275 (6)	0.0032 (4)	0.0004 (4)	0.0059 (4)
Cl2	0.0294 (6)	0.0242 (6)	0.0138 (5)	−0.0038 (5)	−0.0010 (4)	−0.0003 (4)
Cl3	0.0240 (6)	0.0181 (5)	0.0199 (5)	−0.0040 (4)	−0.0016 (4)	0.0032 (4)
N1	0.028 (2)	0.0205 (19)	0.0145 (17)	−0.0016 (16)	−0.0025 (15)	0.0007 (14)
C9	0.035 (3)	0.026 (2)	0.017 (2)	0.007 (2)	0.003 (2)	0.0041 (18)

### Geometric parameters (Å, º)

**Table:**

O1—C1	1.351 (6)	C8—H8B	0.9800
O1—C8	1.438 (6)	C8—H8C	0.9800
O2—C5	1.231 (6)	Sn1—Cl1^i^	2.4100 (12)
C1—C2	1.390 (7)	Sn1—Cl1	2.4100 (12)
C1—C7	1.399 (7)	Sn1—Cl3^i^	2.4220 (11)
C2—C3	1.384 (7)	Sn1—Cl3	2.4220 (11)
C2—H2	0.9500	Sn1—Cl2^i^	2.4322 (11)
C3—C4	1.385 (6)	Sn1—Cl2	2.4322 (11)
C3—H3	0.9500	N1—C9	1.482 (6)
C4—C6	1.395 (7)	N1—H1A	0.902 (19)
C4—C5	1.449 (7)	N1—H1B	0.92 (2)
C5—H5	0.9500	N1—H1C	0.925 (19)
C6—C7	1.386 (7)	C9—C9^ii^	1.490 (10)
C6—H6	0.9500	C9—H9A	0.9900
C7—H7	0.9500	C9—H9B	0.9900
C8—H8A	0.9800		
			
C1—O1—C8	117.8 (4)	Cl1^i^—Sn1—Cl1	180.0
O1—C1—C2	124.8 (4)	Cl1^i^—Sn1—Cl3^i^	90.80 (4)
O1—C1—C7	114.4 (4)	Cl1—Sn1—Cl3^i^	89.21 (4)
C2—C1—C7	120.7 (5)	Cl1^i^—Sn1—Cl3	89.20 (4)
C3—C2—C1	119.1 (4)	Cl1—Sn1—Cl3	90.79 (4)
C3—C2—H2	120.4	Cl3^i^—Sn1—Cl3	180.0
C1—C2—H2	120.4	Cl1^i^—Sn1—Cl2^i^	90.64 (4)
C2—C3—C4	121.2 (5)	Cl1—Sn1—Cl2^i^	89.36 (4)
C2—C3—H3	119.4	Cl3^i^—Sn1—Cl2^i^	89.80 (4)
C4—C3—H3	119.4	Cl3—Sn1—Cl2^i^	90.20 (4)
C3—C4—C6	119.1 (5)	Cl1^i^—Sn1—Cl2	89.36 (4)
C3—C4—C5	120.4 (5)	Cl1—Sn1—Cl2	90.64 (4)
C6—C4—C5	120.5 (5)	Cl3^i^—Sn1—Cl2	90.20 (4)
O2—C5—C4	124.2 (5)	Cl3—Sn1—Cl2	89.80 (4)
O2—C5—H5	117.9	Cl2^i^—Sn1—Cl2	180.0
C4—C5—H5	117.9	C9—N1—H1A	109 (4)
C7—C6—C4	120.8 (4)	C9—N1—H1B	111 (4)
C7—C6—H6	119.6	H1A—N1—H1B	120 (6)
C4—C6—H6	119.6	C9—N1—H1C	110 (4)
C6—C7—C1	119.0 (5)	H1A—N1—H1C	109 (6)
C6—C7—H7	120.5	H1B—N1—H1C	97 (6)
C1—C7—H7	120.5	N1—C9—C9^ii^	110.6 (5)
O1—C8—H8A	109.5	N1—C9—H9A	109.5
O1—C8—H8B	109.5	C9^ii^—C9—H9A	109.5
H8A—C8—H8B	109.5	N1—C9—H9B	109.5
O1—C8—H8C	109.5	C9^ii^—C9—H9B	109.5
H8A—C8—H8C	109.5	H9A—C9—H9B	108.1
H8B—C8—H8C	109.5		
			
C8—O1—C1—C2	0.1 (8)	C3—C4—C5—O2	−179.3 (6)
C8—O1—C1—C7	−179.6 (5)	C6—C4—C5—O2	0.6 (9)
O1—C1—C2—C3	−179.7 (5)	C3—C4—C6—C7	0.6 (9)
C7—C1—C2—C3	−0.1 (9)	C5—C4—C6—C7	−179.4 (5)
C1—C2—C3—C4	0.6 (9)	C4—C6—C7—C1	0.0 (9)
C2—C3—C4—C6	−0.9 (9)	O1—C1—C7—C6	179.5 (5)
C2—C3—C4—C5	179.1 (5)	C2—C1—C7—C6	−0.2 (9)

Symmetry codes: (i) −*x*, −*y*, −*z*; (ii) −*x*+1, −*y*, −*z*+1.

### Hydrogen-bond geometry (Å, º)

**Table:**

*D*—H···*A*	*D*—H	H···*A*	*D*···*A*	*D*—H···*A*
C6—H6···Cl1^iii^	0.95	3.05	3.596 (5)	118
N1—H1*A*···O2^iii^	0.90 (2)	1.89 (3)	2.763 (6)	162 (6)
N1—H1*B*···Cl1^iv^	0.92 (2)	2.71 (5)	3.312 (4)	124 (5)
N1—H1*B*···Cl3^v^	0.92 (2)	2.62 (4)	3.404 (4)	144 (5)
N1—H1*C*···Cl2	0.92 (2)	2.44 (3)	3.315 (5)	158 (6)
N1—H1*C*···Cl3	0.92 (2)	2.75 (6)	3.292 (4)	119 (5)
C9—H9*B*···O2^vi^	0.99	2.62	3.319 (7)	128

Symmetry codes: (iii) *x*+1/2, −*y*+1/2, *z*+1/2; (iv) *x*+1, *y*, *z*; (v) −*x*+1, −*y*, −*z*; (vi) −*x*+1/2, *y*−1/2, −*z*+1/2.
